# The national disability insurance scheme and parenting support for families of children with developmental disability: A need for policy reform

**DOI:** 10.1177/00048674231192369

**Published:** 2023-08-19

**Authors:** Trevor G Mazzucchelli, Bruce J Tonge, Avril V Brereton, Catherine Wade, Kirsten Baird-Bate, Sharon Dawe

**Affiliations:** 1 School of Population Health, Curtin University, Perth, WA, Australia; 2 Parenting and Family Support Centre, School of Psychology, The University of Queensland, QLD, Australia; 3 Centre for Developmental Psychiatry and Psychology, Monash University, Clayton, VIC, Australia; 4 Parenting Research Centre, East Melbourne, VIC, Australia; 5 School of Early Childhood and Inclusive Education, Queensland University of Technology, Brisbane, QLD, Australia; 6 School of Applied Psychology, Griffith University, Brisbane, QLD, Australia

Parents are uniquely placed to provide an optimal environment for their children’s development and benefit from support in doing so. Such support does not need to be intensive, but rather delivered at a critical period of neurodevelopment to maximise potential gains. Parenting programmes for neurodiverse children have been shown to enhance lasting skill development and day-to-day functioning, including communication, play and daily living skills. Parenting programmes have also been shown to reduce behaviours of concern, increase community participation and enhance mental health and wellbeing in both children and parents/carers (Mazzucchelli et al., 2022). In addition to the benefits for both children and carers, funding parenting support makes good economic sense. Parenting programmes for parents of children with a disability save more money than they cost due to the increased capacity of parents to return to work and to provide sustainable support for their children (Einfeld et al., 2018).

The Australian Government’s National Disability Insurance Scheme (NDIS) represents one of the most important social reforms in Australian history. Guided by principles of self-determination, equity and inclusion, this scheme moved away from block funding of disability services to a personalised approach that provides individually tailored packages of services. The intention is to place the person with a disability, or if a child, their parents, at the centre of decision-making process and permit greater choice, control and tailoring of supports. Although this new approach has clear benefits for the person with a disability, there is emerging evidence that there are also risks and inadvertent consequences (Boaden et al., 2021). One such consequence may be a reduction in the provision of specialist parenting support.

Children with a range of neurodevelopmental disabilities (including autism, intellectual disability and foetal alcohol spectrum disorder) are eligible for individual therapy and support under the NDIS. To obtain an optimal mix of supports, the personalised funding model of the NDIS requires extensive coordination and liaison. People with a disability and their families must have the knowledge and capacity to understand and prioritise support needs, make informed decisions regarding which services are evidence-based to best meet these needs, evaluate the quality of services, advocate with an NDIS local area planner for desired services, and access these services. The model also assumes that needed supports are readily available, and that the NDIS planner has sufficient knowledge of which supports are indicated. The shift away from block funding has meant that families are no longer being routinely offered parenting support, and there are growing concerns that parents are no longer aware of the relevance, benefits or how to access tailored parenting support. Children may be disadvantaged in the NDIS system because the benefits to them of parenting support are not recognised. Finally, there is a lack of clarity around the funding options for services that come under the broad umbrella of ‘parenting support’ or ‘parenting programs’.

While there is a clear principle that the NDIS supports early childhood intervention for specialist services to promote children’s development and family and child wellbeing, guidance on whether parenting programmes are supported under the existing NDIS seems contradictory. The only reference to ‘parenting programs’ in the NDIS Guidelines (National Disability Insurance Agency, 2022) comes in a section on what the child protection and family support systems are responsible for (i.e. not the NDIS). However, elsewhere it is stated that training and courses to improve parents/carers skills when these would help meet their child’s goals on the NDIS plan are supported, as are disability-specific parent and carer training programs if the focus is on helping children build skills that have been impacted by their developmental delay. This issue of whether the NDIS is or is not responsible for parenting support is similar to the provision of some individual therapies. For example, some plan managers believe that the NDIS is not responsible for psychology services since these are captured by Medicare, Australia’s universal healthcare insurance scheme, whereas others recognise that there are disability-specific complexities that warrant psychological support.

There are a range of disability-focused parenting programmes that have evidence for children with developmental disabilities, including the Parents under Pressure programme, Signposts, and Stepping Stones Triple P (Doyle et al., 2022; Mazzucchelli et al., 2022). However, the current lack of clarity over whether these programmes can be funded under the NDIS has led to growing concern across a range of advocacy and policy groups. On one hand, it is possible to argue that such programmes provide opportunities to increase understanding challenging behaviour, support alternative adaptive behaviour and increase family wellbeing (among other goals), and that this clearly falls within the remit of the NDIS; on the other hand, programmes that are classified as ‘parenting programs’ would appear to be excluded.

In addition to problems with the interpretation of NDIS policy, other barriers also exist. The NDIS price guide gives a large financial incentive for practitioners to provide individual direct services to the child rather than training to carers/parents (https://planpartners.com.au/tools/ndis-price-guide). Furthermore, compared to a parenting programme delivered to an individual family, more cost-effective group programmes, which were previously delivered by a disability service, are less likely to be funded because these now require various parents/caregivers to contemporaneously request the same service.

At present, a key opportunity to enhance enduring lifelong skills for children with developmental disabilities is likely being missed. We argue that various reforms to policy and practice should be introduced that will increase knowledge of, access to, and participation in evidence-based parenting programmes. Specifically, we recommend that:

- The NDIS provide a list of parenting programmes that are considered evidence-based best-practice.
- Purveyors develop and evaluate low-intensity disability-specific parent support options (including online options) to facilitate equitable (and affordable) reach of parenting support to families living in rural and remote areas.
- Multidisciplinary/specialist developmental assessment of form and severity of disability conducted by health professionals, include recommendations regarding the best evidence-based approaches to support the child’s development including access to the most suitable parenting programmes.
- Parents/carers are fully informed by the assessment team of current appropriate parenting programmes in order that they can request them when meeting their NDIS local area coordinator/planner.
- Specialist assessment recommendations regarding a parenting programme are acted upon by the NDIS service planner.
- Training for carers/parents in evidence-based parenting programmes and parent-mediated interventions are remunerated at the same rate as capacity building supports delivered individually to a person with a disability.
- The NDIS provide aggregate data each year on the specific funded services and supports, including parenting programmes, in order to assess the impact of the above recommendations.

These initiatives, if incorporated into NDIS policy, would help to optimise the development of children with developmental disability and reduce family stress.

Evidence-based parenting support should be available to families, but this should not be at the cost of other supports. Although increasing access to evidence-based parenting support through the NDIS is likely to increase the total cost of services in the short-term, we believe it is likely to save taxpayer money in the longer term since it represents an investment in children’s most important and enduring supports – their parents. Increasing parents’ capacity to provide enduring high-quality support, and building independence skills in their children, is likely to be part of the solution to ensure the equitable sustainability of the NDIS.

## Supplemental Material

sj-docx-1-anp-10.1177_00048674231192369 – Supplemental material for The national disability insurance scheme and parenting support for families of children with developmental disability: A need for policy reform Click here for additional data file. Supplemental material, sj-docx-1-anp-10.1177_00048674231192369 for The national disability insurance scheme and parenting support for families of children with developmental disability: A need for policy reform by Trevor G Mazzucchelli, Bruce J Tonge, Avril V Brereton, Catherine Wade, Kirsten Baird-Bate and Sharon Dawe in Australian & New Zealand Journal of Psychiatry
