# Supplementary material for: The national disability insurance scheme and parenting support for families of children with developmental disability: A need for policy reform
Source: Aust N Z J Psychiatry. 2023 Aug 19;57(12):1508–10. doi: 10.1177/00048674231192369 (PMC10666470; doi:10.1177/00048674231192369)
Supplement: sj-docx-1-anp-10.1177_00048674231192369 – Supplemental material for The national disability insurance scheme and parenting support for families of children with developmental disability: A need for policy reform [file sj-docx-1-anp-10.1177_00048674231192369.docx]

**Supplementary Material: Information Relating to Parenting Programs from Australia’s National Disability Insurance Scheme (NDIS) Guidelines**

| **Website** | **Link** | **Evidence** | **Implication/Conclusion** |
| --- | --- | --- | --- |
| NDIS: Reasonable and Necessary Supports | <https://ourguidelines.ndis.gov.au/how-ndis-supports-work-menu/reasonable-and-necessary-supports>  https://www.family-advocacy.com/assets/Uploads/NDIS-Information-Update/798e63c92a/NDIS_Reasonable_and_necessary_flowchart.pdf | “All NDIS supports need to meet all the reasonable and necessary criteria.   - The support will assist you to [pursue your goals in your plan](https://ourguidelines.ndis.gov.au/how-ndis-supports-work-menu/reasonable-and-necessary-supports/how-we-work-out-if-support-meets-funding-criteria/does-support-meet-reasonable-and-necessary-criteria/does-support-help-you-pursue-your-goals). - The support will assist you to [undertake activities, to facilitate your social and economic participation](https://ourguidelines.ndis.gov.au/how-ndis-supports-work-menu/reasonable-and-necessary-supports/how-we-work-out-if-support-meets-funding-criteria/does-support-meet-reasonable-and-necessary-criteria/does-support-help-you-do-activities-will-help-your-social-and-economic-participation). - The support represents [value for money](https://ourguidelines.ndis.gov.au/how-ndis-supports-work-menu/reasonable-and-necessary-supports/how-we-work-out-if-support-meets-funding-criteria/does-support-meet-reasonable-and-necessary-criteria/support-value-money) - The support will be, or is likely to be, [effective and beneficial](https://ourguidelines.ndis.gov.au/how-ndis-supports-work-menu/reasonable-and-necessary-supports/how-we-work-out-if-support-meets-funding-criteria/does-support-meet-reasonable-and-necessary-criteria/support-effective-and-beneficial) for you, having regard to current good practice. - The funding of the support takes account of [what it is reasonable to expect families, carers, informal networks and the community to provide](https://ourguidelines.ndis.gov.au/how-ndis-supports-work-menu/reasonable-and-necessary-supports/how-we-work-out-if-support-meets-funding-criteria/does-support-meet-reasonable-and-necessary-criteria/support-something-we-would-expect-your-informal-supports-provide). - The support is [most appropriately funded or provided through the NDIS](https://ourguidelines.ndis.gov.au/how-ndis-supports-work-menu/reasonable-and-necessary-supports/how-we-work-out-if-support-meets-funding-criteria/does-support-meet-reasonable-and-necessary-criteria/support-more-appropriately-funded-or-provided-through-ndis), and is not more appropriately funded or provided through other general systems of service delivery.” | - NDIS decision making hinges on whether a support is “reasonable and necessary.” A strong argument can be mounted that evidence parenting support meets these criteria (although some might argue that these supports could be provided through other systems of service delivery). |
| NDIS: Reasonable and Necessary Supports | <https://ourguidelines.ndis.gov.au/how-ndis-supports-work-menu/reasonable-and-necessary-supports> | “We only fund supports that will be, or are likely to be, effective and beneficial for you, having regard to current good practice.^i^ This means we consider if there is evidence that the support is effective and beneficial for someone with similar disability support needs.  We will consider different types of evidence when making decisions and we won’t need an expert report for every support, as we can often rely on other information or evidence.  For example, we may already have information about whether the support is widely accepted to suit someone with your disability support needs.^ii^ The primary source of evidence we rely on, and give the greatest weight to, is evidence from sources that are reliable and widely-recognised. This includes published and refereed literature, and any consensus of expert opinions. If there is no evidence to show a support is reasonable and necessary,^iii^ we won’t fund the support.  You can find the types of evidence we need on [our website](https://www.ndis.gov.au/applying-access-ndis/how-apply/information-support-your-request/types-disability-evidence), and in [Our Guidelines](https://ourguidelines.ndis.gov.au/).” | - Further clarification may be required on how evidence-based best practice is judged. What constitutes “consensus of expert opinions”. |
| NDIS: Is the support something we would reasonably expect your informal supports, like family or friends, to provide? | https://ourguidelines.ndis.gov.au/how-ndis-supports-work-menu/reasonable-and-necessary-supports/how-we-work-out-if-support-meets-funding-criteria/does-support-meet-reasonable-and-necessary-criteria/support-something-we-would-expect-your-informal-supports-provide#if-you-re-under-18 | “If you’re under 18, we consider what support is reasonable to expect parents to provide at your age. It’s normal for parents to provide substantial care and support for children. We consider that it’s usual for parents to provide almost all the care and support that young children need.  For example, it’s reasonable to expect parents to provide transport to and from their child’s after-school activities. Of course, the amount of care and support for a child without a disability would typically reduce as they get older.  For children under 18, we consider:   - if your needs are ‘substantially greater’ because of your disability, compared to other children the same age – that is, you need much more disability support - any risks to the wellbeing of people providing informal support to you   if including funding for the support will help build your skills and capacity in the future, or reduce any risks to you.” | - The NDIS recognises parents’ important role for children and considers whether funding for additional supports would enhance the child’s skills and capacity in the future. |
| NDIS: Child protection and family support | https://ourguidelines.ndis.gov.au/how-ndis-supports-work-menu/mainstream-and-community-supports/who-responsible-supports-you-need/child-protection-and-family-support | “Child protection and family support systems are responsible for…. General parenting programs, counselling or other supports for families at risk of entering the child protection system, and to the broader community – this includes adjusting these programs to make them suitable for families with disability.” | - The NDIS is not responsible for parenting programs. |
| NDIS: Support for carers from other agencies | https://www.ndis.gov.au/understanding/families-and-carers/support-carers-other-agencies | Provides links to Department of Social Services and Carers Gateway. These services are focused on the health and wellbeing of carers rather than the provision of parenting programs. | - Support for carers is available through other agencies (e.g., Department of Social Services, Carers Gateway), but this does not extend to parenting programs. |
| NDIS: How we help carers | https://www.ndis.gov.au/understanding/families-and-carers/how-we-can-help-carers | “In addition to the government-funded programs available to support carers, a NDIS participant might use funding in their plan to facilitate respite.”  “For carers, taking some time off can help them better manage their own health and improve their wellbeing  In deciding whether to fund or provide a support, we will take into account what is reasonable to expect from families, carers, informal networks and the community.” | - The NDIS may fund supports (including respite), but the emphasis remains on the health and wellbeing of carers rather than enhancing skills through parenting programs. |
| NDIS: Early childhood development | https://ourguidelines.ndis.gov.au/how-ndis-supports-work-menu/mainstream-and-community-supports/who-responsible-supports-you-need/early-childhood-development#wesupportexamples | “We’re responsible for supports that are specific to a child’s developmental delay or disability. This might be if a child needs more support than other children of a similar age, and more support than what early childhood services must provide as a reasonable adjustment.  This includes early intervention supports that children need to help build their skills, because of their developmental delay or disability. We may fund supports that are both:   - focused on improving a child’s daily living skills, such as communicating with those around them, participating in social activities, and completing self-care tasks such as dressing - likely to increase a child’s independence and reduce how much NDIS support a child needs in the future, for example with a series or mix of different supports. - Remember, if we’re responsible for funding the support, it must also meet all the other [NDIS funding criteria](https://ourguidelines.ndis.gov.au/how-ndis-supports-work-menu/reasonable-and-necessary-supports/how-we-work-out-if-support-meets-funding-criteria) before we can include it in your plan.” | - The emphasis in early childhood development is on communication, daily living and increasing independence. For parenting programs to be funded, there may need to be an argument as to how these help promote these skills. |
| NDIS: Training for Carers/Parents | https://planpartners.com.au/tools/ndis-price-guide/15_038_0117_1_3 | This funding is for parents/carers in matters relating a person’s disability and how to care for them.  Price varies from $70.87/hour to $106.31/hour depending on level of remoteness. | - Funding for training for carer/s parents is substantially less than funding for direct contact with children. |
| NDIS: Early childhood Supports--Psychologist | https://planpartners.com.au/tools/ndis-price-guide/15_001_0118_1_3#content | This is funding for Psychologist for children under 5 years of age  Prices varies from 214.41/hour to $352.25/hour depending on location. | - Funding for training for carer/s parents is substantially less than funding for direct contact with children. |
| NDIS: Early Childhood Supports—Early Childhood Professional | https://planpartners.com.au/tools/ndis-price-guide/15_005_0118_1_3#content | This is funding for Therapies (excluding Physiotherapy and Psychology) for children under 5 years of age for example: OT, occupational therapy, Speech, speech therapy consult, consultation, therapies, pediatry, chiropractor.  Prices varies from 193.99/hour to $290.99/hour depending on location. | - Funding for training for carer/s parents is substantially less than funding for direct contact with children. |
| Children and Young People with Disability (CYDA) | https://www.cyda.org.au/images/pdf/icw5_parent_and_carer_training.pdf | “As a parent or carer of your child or young person with disability, you can receive funding to improve your ability to support them to achieve their goals…. Some examples of training for your caring role include:…   - Understanding challenging behaviour - Supporting positive behaviour   …The NDIS Price Catalogue has a specific item for parent and carer training and it is a good idea to mention your need for training during your planning meeting.” | - Parent and carer training can be funded through the NDIS, although parenting programs are not specifically mentioned, and parents may need guidance to advocate for these services. |

*Note***.** All information accessed on 13 December 2022. iNDIS Act s 34(1)(d). i^i^NDIS (Supports for Participants) Rules r 4.1(d). i^ii^NDIS Act s 34(1).

**Personal Communication from K. Baird-Bate on 24 November 2022**

- A contact reported: what is funded by NDIS appears inconsistent across Australia and in different regions.

This is consistent with reports I have had from others. For instance:

- A contact (not a psychologist) works at a NDIS funded organisation that helps parents of children with autism implement therapy supports. While this service is funded, the “parent coaching” service they also offer is not NDIS funded.
- Two other contacts (one a registered psychologist) have run parenting programs that are funded by the NDIS

**Personal Communication from A. Brereton on 24 November 2022**

Although there are excellent evidence-based parenting training programs for families who have an autistic child, programs that are being offered as parent education/training and funded by the NDIS include inTune Families, Autism360, Autism Parental Stress Relief Program, and Reframing Autism. It is not clear these have accumulated good evidence to support them.
